# How Is Variety in Daily Life Related to the Expression of Personality States? An Ambulatory Assessment Study

**DOI:** 10.1177/08902070221149593

**Published:** 2023-01-09

**Authors:** Stefanie Lindner, Mirjam Stieger, Dominik Rüegger, Tobias Kowatsch, Christoph Flückiger, Matthias R. Mehl, Mathias Allemand

**Affiliations:** 1 27217 University of Zurich, Zurich, Switzerland; 2 Lucerne University of Applied Sciences and Arts, Lucerne, Switzerland; 3 ETH Zurich, Zurich, Switzerland; 4 University of St Gallen, St.Gallen, Switzerland; 5 University of Arizona, Tucson, AZ, USA; 6 University of Kassel, Kassel, Germany

**Keywords:** variety in daily life, expression of personality states, within-person variability, ambulatory assessment, daily life research

## Abstract

People differ in the way they live their daily lives. For some people, daily life is characterized by multiple and diverse experiences, while others have more stability and routine in their lives. However, little is known about how variety in daily life relates to the expression of personality states. The present study examined within-person associations between variety in social partners, places, and activities with state expression. Data came from an ambulatory assessment study (*N* = 962, *M*_age_ = 25.49) with four assessments per day over a period of six consecutive days. The results of the multilevel modeling analyses suggest that variety in daily life is associated with some, but not all, state expressions. For instance, on days when participants experienced a greater variety in activities, they reported being less neurotic and conscientious, but also more agreeable. In addition, the links between all social partners, places, and activities with the expression of the state were examined simultaneously to obtain more detailed information on the multifaceted nature of situation-state expression links. We conclude that variety in daily life has both theoretical and empirical relevance for the expression of personality states.

## Introduction

People differ in the way they live their daily lives. Some people feel comfortable following a daily routine, while others enjoy a diverse lifestyle that allows them to meet different people (Fingerman et al., 2020; Zhaoyang et al., 2018), move frequently from place to place (Matz & Harari, 2021; Weber et al., 2020), and engage in a wide array of activities (Jackson et al., 2020; Lee et al., 2018). These individual differences can provide opportunities and constraints for the expression of personality states, that is, how people express their behavior, thoughts, and feelings at a particular time and situation in a way that is consistent with their personality traits (Baumert et al., 2017; Fleeson, 2001; Wrzus & Roberts, 2017). Previous work has focused on associations between the expression of personality states and social experiences with different social partners (Breil et al., 2019; Wrzus et al., 2016), places visited (Matz & Harari, 2021), activities (Wrzus et al., 2016), and perceptions of situation characteristics (Noftle & Gust, 2019; Rauthmann et al., 2016). Other studies examined personality traits and situational experiences as predictors of state expression (Sherman et al., 2015), while others examined how personality traits contribute to associations between social partners, activities, and affective experiences (Wilt & Revelle, 2019). However, few (if any) naturalistic studies have examined *variety* in social partners, places visited, and activities simultaneously in relation to state expression. The aim of the present study was thus to examine how variety in daily life is related to the expression of personality states.

### Variety in Daily Life Between and Within Individuals

In this study, we focus on three *situation cues*: (a) social partners (i.e., with whom time is spent; e.g., partner, friends, or being alone), (b) places (i.e., where time is spent; e.g., being at home, at the workplace, or outdoor place), and (c) activities (i.e., what one is doing; e.g., working, relaxing, holding a conversation). Situation cues are defined as physical and objectively quantifiable stimuli of a situation (e.g., whether other people are present) that can be perceived and interpreted by people, resulting in psychological situation characteristics (e.g., whether work needs to be done; how positive or stressful a situation is) (Rauthmann, 2015; Rauthmann et al., 2015). While several taxonomies of major dimensions of psychological situation characteristics exist (e.g., Situational Eight DIAMONDS; Rauthmann et al., 2014), replicable structures or lists of situation cues do not yet exist (Rauthmann & Sherman, 2020; but see Noftle & Gust, 2015 for a suggestion). Although the three above-mentioned situation cues do not comprise a comprehensive taxonomy, they do cover a wide range of social partners, places, and activities (e.g., Noftle & Gust, 2015; Rauthmann, 2015). In this study, we use the term *variety in daily life* to describe the number of individual categories related to social partners, places, and activities during or across days (e.g., Benson et al., 2018; Noftle & Gust, 2015; Weber et al., 2020)^1^. For example, some people have daily interactions with different social partners and therefore have a greater variety of social partners, while others tend to always meet the same social partners and thus have a low variety in social partners. Although there are often numerous opportunities in daily life to meet different people, visit different places, and participate in a variety of social activities, not everyone takes advantage of these opportunities. Note that variety is bounded by the total number of categories observed or assessed for each of the situation cues.

Variety in daily life can be examined from at least two perspectives. From a between-person perspective, people may differ from one another in the ways their lives are shaped by variety, suggesting variety *between* individuals. For instance, some people generally prefer a highly structured, stable, and routinized daily life that is more homogeneous and less diverse. In contrast, other people allow or strive for more flexibility in their daily lives and may therefore be exposed to a wider array of people, places, and activities. Variety may be influenced by situational opportunities and constraints. For example, some jobs allow flexible time management (possibly offering more opportunities for variety), while others necessitate a more rigid schedule (likely limiting opportunities for variety). Previous research has shown that people differ in how diverse their daily lives are in terms of social partners, places, or activities (Fingerman et al., 2020; Jackson et al., 2020; Sandstrom et al., 2017). For example, recent studies found that variety in social partners was positively related to variety in activities (Fingerman et al., 2020) and places (Weber et al., 2020). Interactions with different social partners often involve a variety of activities and take place in different locations. Furthermore, a recent longitudinal study shows that it is unlikely to visit the same place twice unless the place is one’s own home, and that individuals visit more places on a weekday than at the weekend (Servia-Rodriguez et al., 2017).

From a within-person perspective, people may also differ in how their daily lives play out from day to day, indicating variety *within* individuals. For instance, some people pursue a more diverse lifestyle than others, but their tendency to do so may vary from day to day. On one day, people may intentionally or unintentionally pursue different activities and take advantage of opportunities for diverse activities, while on other days, they may pursue similar activities due to lack of time and other constraints. However, one caveat of previous research is that it has primarily focused on between-person analyses of variety (Lee et al., 2018; Weber et al., 2020; Zhaoyang et al., 2018) and thus has neglected the potential for *within-person variety*. It is a largely open question how people differ from day to day in variety in social partners, places, and activities.

### Variety in Daily Life and Expression of Personality States

Personality traits are defined as relatively enduring patterns of behavior, thoughts, and feelings (Roberts & Jackson, 2008). Despite their relatively enduring nature, their expression may fluctuate from moment to moment and from situation to situation, respectively. We use the term *state expression* to describe the extent to which people express their behavior, thoughts, and feelings at a particular time and situation in a way that is consistent with their personality traits (Baumert et al., 2017; Fleeson, 2001; Wrzus & Roberts, 2017). Numerous studies found substantial variation or fluctuations in the expressions of personality states over repeated assessments across situations (Church et al., 2013; Fleeson, 2001; Fleeson & Gallagher, 2009; Lindner et al., 2021, 2022; Noftle & Fleeson, 2010). Hence, variety in daily life may be responsible, in part, for variation in state expression.

Several theoretical accounts provide guidance to the question of how variety in daily life may relate to the expression of personality states (Buss, 1987; Fleeson & Jayawickreme, 2015; Jayawickreme et al., 2019; Rauthmann, 2021; Wrzus & Roberts, 2017). The state expression can be viewed as a function of situational factors including variety in daily life and individual influences. For example, the TESSERA model suggests that both situation cues and psychological situation characteristics directly trigger state expressions, or indirectly by triggering expectations about how one should behave, think, or feel in the given situation (Wrzus & Roberts, 2017). Similarly, Whole Trait Theory (WTT; Fleeson & Jayawickreme, 2015; Jayawickreme et al., 2019) posits that state expressions are driven by situational factors and individual processes, such as cognitive processes (e.g., perceiving and interpreting a situation in a way that promotes neurotic behaviors) or motivational processes (e.g., pursuing the goal of being the center of attention may lead to more extraverted behaviors). Furthermore, how people perceive affordances of situations can facilitate state expressions in a given situation (Church et al., 2010; Fleeson & Noftle, 2008; Noftle & Gust, 2019). For example, hanging out in a bar with friends affords extraverted behavior, whereas working in an office affords more conscientious behaviors compared to leisure activities. Hence, variety in social partners, places, and activities may call for greater variation in how people express their personality states to navigate situations and behave in accordance with perceived situational affordances (Fleeson & Jayawickreme, 2015; Jayawickreme et al., 2019; Kashdan & Rottenberg, 2010).

Variety in daily life can be seen to some extent as a function of how people express their personality states by moving into, influencing, and shaping situations. For instance, people may actively seek out or avoid situations to elicit a particular state expression (e.g., visiting a library instead of a bar to behave conscientiously), actively modify a given situation (e.g., extraverted behavior may encourage others to behave sociably), or passively elicit responses from others (e.g., pleasant behavior may elicit warm and considerate social responses from others) (Buss, 1987; Rauthmann, 2021; Rauthmann & Sherman, 2020). The directionality of the association between state expression and situation cues may be bidirectional such that changes in situation cues may precede and succeed changes in state expression (Rauthmann & Sherman, 2020). Examination of the directionality between psychological situation characteristics and state expression using experience sampling methods has shown little spillover from situational experiences to personality states and vice versa (Sherman et al., 2015).

Although the association between situation cues and state expression is at the core of the person-situation literature (Baumert et al., 2017; Fleeson & Jayawickreme, 2015; Jayawickreme et al., 2019; Mischel & Shoda, 1995; Rauthmann, 2021; Rauthmann & Sherman, 2020), little is known about how variety in daily life is related to state expression. Previous research has mainly focused on individual situation cues (e.g., social partners or places or activities) in relation to state expression (Breil et al., 2019; Fleeson, 2001, 2007; Matz & Harari, 2021; Wilson et al., 2017), neglecting the fact that daily life is typically characterized by multiple situation cues at the same time (e.g., social partners and places and activities, etc.). In addition, most previous studies have not examined variety in daily life across and within days (but see Weber et al., 2020). Finally, with some exceptions (e.g., Matz & Harari, 2021), most existing studies have included relatively small samples, which can limit the robustness of the findings. Accordingly, studies of variety in social partners, places, and activities are urgently needed to better understand how individual differences in how people live their lives contribute to the expression of personality states in individuals.

### Social Partners and Expression of Personality States

A greater variety in social partners can provide opportunities to share social support, develop and manage a social identity, and to manage conflict (Leary, 2007), for which state expression may be adaptive. For instance, one can mirror communal behaviors (e.g., show extraversion when the partner acts extraverted), or enact complementary behaviors (e.g., respond with dominant behavior when the partner shows submissive behavior) to connect with others or to control a situation (Brumbaugh & Wood, 2013; Moskowitz et al., 2007; Yao & Moskowitz, 2015). As a consequence, people may vary in their state expression across social partners to adjust to a given situation. In contrast, a greater variety in social partners can also be a source of stress for less extraverted individuals, as they have to adapt to a wide range of social interaction partners.

Borrowing from the trait literature, individuals report different levels in personality traits when they are with different people (e.g., with parents or friends; Robinson, 2009; Sheldon et al., 1997). A study in the workplace found that employees varied in their state expression when they were among different people: Employees displayed lower extraversion, agreeableness, and conscientiousness when interacting with their supervisor or peers than when interacting with customers (Huang & Ryan, 2011). Previous research suggests that the company of people provides opportunities to show more extraverted behaviors (Breil et al., 2019; Geukes et al., 2017; Rauthmann et al., 2016). Based on these findings, one would expect that a greater variety in social partners may promote greater state expression, especially extraverted behavior. Previous studies did not distinguish between the variety in social partners in daily life, such as partners, friends, or strangers. These studies did not simultaneously consider multiple situation cues, such as social partners and places visited.

### Places and Expression of Personality States

Personality psychologists have become increasingly interested in linking places to personality (Graham et al., 2015; Matz & Harari, 2021; Mehl et al., 2006; Tackman et al., 2020). Places represent certain traditions and lifestyles and can be useful for everyday practices influencing where people spend their time and lives (Graham et al., 2015; Krämer, 1996). Previous studies found that people with higher levels of extraversion spent more time closer to the campus center (Oishi & Choi, 2020), and people with higher levels of agreeableness and conscientiousness were more often in public places and less often inside their apartments (Mehl et al., 2006; Tackman et al., 2020). However, these studies focused on overall trait levels and not state expression.

There are few studies on the association between places and state expression, and the findings are mixed (Geukes et al., 2017; Matz & Harari, 2021; Sandstrom et al., 2017). One study found that interpersonal behaviors vary across different places (e.g., at the university, outside, at home), but this study did not examine specific place-state relationships (Geukes et al., 2017). Another study with a small sample size (*N* = 69) and a focus on three specific places (home, work, social place) found scant evidence for links between these places and state expression (as reported in the Supplemental material of Sandstrom et al., 2017). In contrast, recent large-scale ambulatory assessment studies with large sample sizes (*N*’s = 446 to 1196) and a broad and diverse range of places (e.g., bar/party, gym, library) showed that places significantly contributed to variation in state expression (Matz & Harari, 2021). For instance, places had the strongest effect on extraversion and the least effect on neuroticism. Places were also concurrently associated with activities performed either alone or with others, all of which can simultaneously influence state expression. To date, little research has examined how different places are related to the expression of states when a variety in social partners and activities are considered simultaneously.

### Activities and Expression of Personality States

A typical day consists of various activities, such as working, doing chores, participating in leisure activities, and socializing. Activities come with affordances that may offer opportunities and exert constraints on certain state expressions. To adapt to the demands of activities, people can adjust accordingly and vary how they express states (Paulus & Martin, 1988). Engaging in diverse activities may thus elicit opportunities and constraints for state expression. Indeed, previous work has shown that activities are associated with certain state expressions (Aschwanden et al., 2018; Beckmann et al., 2020; Fleeson, 2007; Minbashian et al., 2010; Rauthmann et al., 2016; Wilson et al., 2017; Wood et al., 2019). For instance, laboratory studies found that people vary in their state expressions across job-related activities (Beckmann et al., 2020). Findings from ambulatory assessment studies suggest that working and studying are linked with more neurotic and conscientious behaviors (Minbashian et al., 2010; Wilson et al., 2017). Moreover, engaging in duty-related activities (e.g., sending a letter) is associated with more conscientious behaviors (Rauthmann et al., 2016). In contrast, involvement in cognitive activities (e.g., watching a documentary film) is linked with more open behaviors (Aschwanden et al., 2018). However, little attention has been paid to how variety in other important daily activities such as sports, media consumption, or relaxation relates to state expression when different social partners and places visited are also considered.

### Present Study

The purpose of the present study was to investigate how variety in daily life is related to the expression of personality states by examining variety in terms of social partners, places, and activities. The study was guided by four research questions: (1) How diverse is daily life in terms of social partners, places, and activities? We investigated variety in two ways. First, we used a *between-person* approach and examined how people differ from one another in the ways their lives are shaped by variety. Following previous research (e.g., Zhaoyang et al., 2018), we investigated the average variety across six days*.* Second, we used a *within-person* approach to explore how people’s daily lives fluctuate in variety from day to day (daily variety). (2) How variable is the expression of personality states in daily life? Based on previous work on state expression (Fleeson & Gallagher, 2009; Lindner et al., 2022; Wilson et al., 2017), we expected substantial within-person variation in state expression. (3) How is variety in daily life related to the expression of personality states? We used a *bivariate* approach to examine how variety in daily life was related to state expression. Based on theoretical assumptions that encountering different situations contributes to within-person variation in state expression (Fleeson & Jayawickreme, 2015; Wrzus & Roberts., 2017), we expected that variety in daily life is associated with state expression. (4) How are social partners, places, and activities related to the expression of personality states? We used a *multivariate* approach to examine how all individual categories of social partners, places, and activities were simultaneously related to state expression to better understand the unique effect of each situation feature. This approach allows to capture the concept of “variety” using a different analytical approach.

## Methods

### Participants

Participants (*N* = 962) came from the PEACH (PERsonality coACH) study (Stieger et al., 2021).^2^ Eligibility requirements for study participation included fluency in German, being at least 18 years old, and absence of mental health disorders and other psychosocial problems. Participants were primarily recruited via university mailings and social media advertisements. Additionally, participants responded to flyers or word-of-mouth recruitment. The initial sample consisted of 1523 participants. The final sample of individuals who participated in the daily assessments included *N* = 962 (47.8% male) and ranged in age from 18 to 69 years (*M* = 25.49, *SD* = 7.35). Regarding highest educational qualification, most of the participants indicated high school (45%), followed by 21% and 16% who had a bachelor’s and master’s degree, respectively. Seven percent indicated apprenticeship, 5% university of applied sciences, 4% secondary school, and 2% PhD. Most of the participants were in a relationship (48%), 42% were single, 7% were married, 1% were divorced or separated, and 2% indicated others.

We conducted attrition analyses by comparing the demographic variables of the participants included in this study with those individuals who did not participate in the daily assessments, respectively (*n* = 563). The average age of individuals who dropped out was 24.46 years (*SD* = 6.22) compared to 25.50 years (*SD* = 7.35) among completers. This small age difference was significant, *t* (1664) = 3.54, *p* < .00, Cohen’s *d* = .06. The gender balance was comparable between the dropouts (47.7% male) and the final sample (47.9% male), χ^2^ (1) = .023, *p* = .88.

### Study Design and Procedure

The PEACH study consisted of three phases including (a) a pre-intervention phase, (b) an intervention phase, and (c) a post-intervention phase (Stieger et al., 2021). All participants started the study between April 2018 and August 2018. We used the data from the pre-intervention phase before any intervention had taken place.^3^ The study was approved by the Ethics Committee of the Philosophical Faculty of the University of Zurich (No. 17.8.4; date of approval: August 31, 2017). Participants installed the PEACH application on their smartphones (Android or iOS) and gave written informed consent after a detailed explanation of the study. During the pre-intervention phase, participants were asked to behave as usual and not to change their daily lives in order to measure their baseline behavioral signatures. Starting on a Monday, participants responded four times per day to ambulatory assessment questionnaires across six consecutive days. On each day, four assessments were randomly timed within a fixed time window (9:30 a.m.–11:30 a.m., 12:30 p.m.–14:30 p.m., 15:30 p.m.–17:30 p.m., and 18:30 p.m.–20:30 p.m.). Participants received a push-notification on their smartphone prompting them to respond to questions about cues of situations and state expression. Within the time windows, the push-notification was displayed on the smartphone until the questions were responded to. From a total of 23,088 potential observations (962 participants × 4 daily assessments × 6 days), we had between 12,845 and 14,343 observations depending on the variable of interest (on average 67.5%). On average, each participant provided 14.1 surveys out of 24 possible surveys.

### Measures of Personality States

State expression was assessed four times per day. Each Big Five dimension was measured with two bipolar adjective items (neuroticism: “tense – relaxed,” “not confident - self-confident”; extraversion: “shy – outgoing,” “quiet – talkative”; openness: “uninterested – curious”, “narrow-minded – open-minded”; agreeableness: “insensitive – empathic,” “distrustful – trusting”; conscientiousness: “imprudent – deliberate,” “not conscientious – conscientious”). Items were inspired by previous research (Fleeson, 2001, 2007; Noftle & Fleeson, 2010). The adjectives were presented in random order. Participants rated the degree to which each item described them during the last 30 minutes on a slider ranging from 1 (*disagree strongly*) to 100 (*agree strongly*). The scores were converted into a more intuitive 7-point Likert-type scale ranging from 1 to 7 to allow descriptive comparisons with other studies (e.g., Fleeson, 2001; Fleeson & Gallagher, 2009; Sherman et al., 2015).^4^ Estimates of reliability of within-person change (Shrout & Lane, 2012) ranged from .72 (openness) to .83 (extraversion), suggesting that the two-item measures for each state expression scale assessed within-person variability with moderate to substantial reliability.

### Measures of Situation Cues

#### Social Partners

Four times per day, participants indicated *with whom* they are at the moment, choosing one of eight response categories (1 = *alone* (nobody), 2 = *partner*, 3 = *family/children*, 4 = *friends*, 5 = *colleagues,* 6 = *acquaintances*, 7 = *strangers*, 8 = *other category [please specify]*). Categories of social partners were based on previous work (Weber et al., 2020; Wrzus et al., 2016) and reflect a broad range of interaction partners. In 1.1% cases, participants indicated “others” and specified the person. These cases were excluded from the analysis.

#### Places

Four times per day, participants indicated *where* they are at the moment, choosing one of seven response categories (1 = *indoor at home*, 2 = *indoor at workplace*, 3 = *indoor at a private place*, 4 = *indoor at a public place*, 5 = *outdoor at a private place*, 6 = *outdoor at a public place*, 7 = *in transit*). These places were intended to cover a wide array of everyday locations and were similar to the categories used by Weber et al. (2020).

#### Activities

Four times per day, participants reported *what* they are doing at the moment. Participants could choose one of eight response categories (note that some categories included multiple similar activities) (1 = *working, studying, volunteering*, 2 = *chores, errands*, 3 = *sport, movement*, 4 = *media*, 5 = *doing nothing, relaxing, sleeping*, 6 = *conversation, visit*, 7 = *leisure*, 8 = *other category [please specify]*). These activities were based on previous work (Geukes et al., 2017; Weber et al., 2020; Wrzus et al., 2016) and covered a broad range of daily activities (e.g., Rauthmann, 2015). In 6.5% of the cases, participants chose the category “others” and specified the activity. These cases were relatively heterogeneous (e.g., “vote”, “car breakdown”) and did not fit into the existing categories and were thus excluded from the analysis.

### Statistical Analyses

The analyses were exploratory, not preregistered, and performed in four steps, with each step addressing one of the four research questions. First, following Weber et al. (2020), we computed variety scores for social interaction partners, places, and activities separately by (a) averaging the respective indicators per domain across six days to investigate between-person differences in *average variety*, and (b) within days (i.e., how social partner, places, and activities varied between the four assessments per day) to examine within-person differences in *daily variety* from day to day. A variety index quantifies the range and uniformity of involvement in daily life (e.g., Benson et al., 2018). It refers to the number of different categories related to each of the three situation cues. Moreover, the focus here is on variety *between* categories within each of the situation cues. It does not capture variety *within* each category, as some categories have by definition a broader range. For example, whereas the social partners category “partner” typically refers to one particular person, the category “colleagues” may comprise several different people with whom one may have very different relationships (e.g., superiors and peers).

Following previous research (e.g., Benson et al., 2018; Weber et al., 2020; Zhaoyang et al., 2018), we computed a *variety index* (also called diversity index; Shannon, 1948) as follows

$${variety}_{i}=-\left(\frac{1}{\ln\left(m\right)}\right)\overset{m}{\underset{j=1}{\sum }}p_{ij}\ln\left(p_{ij}\right)$$

where *m* is the total number of categories of social partners, places, or activities (e.g., *m* = 7 social partners, *m* = 7 places, *m* = 7 activities), *p*_*ij*_ is the proportion of individual *i*’s total frequency of social partners *j,* total frequency of place *j*, or total frequency of activity *j* across six days (i.e., one score per participant for *average variety* in social partners, places, and activities), and within per day (i.e., one score per participant per day for the *daily variety* in social partners, places, and activities), respectively (*j* = 1 to *m*). Higher variety scores indicate that an individual has spent time with many different social partners, was at many different places, or did many different activities either across the entire study period (average variety), or throughout a day (daily variety).

Second, we computed within-person mean (i*M*) and within-person variability (i*SD*) scores for personality state expression. Additionally, we estimated unconditional random-intercept-only multilevel models (Bolger & Laurenceau, 2013) without predictor variables to calculate the intraclass correlation coefficients (ICCs) to estimate between-person differences in proportion to within-person variation in state expression.

Third, to examine within-person associations between variety in daily life and state expression, we used multilevel modeling (Bolger & Laurenceau, 2013) with observations (Level 1) being nested within participants (Level 2). The data structure consisted of 24 measurement points (maximum number of possible responses) per person. We analyzed five random-intercept-random-slope models with state expression for each of the Big Five domains as the respective outcome. As predictor variables, we included daily variety indexes of social partners, places, and activities simultaneously in the models. Each predictor variable was split into a sample-mean-centered component and a person-centered component to control for the between-person effects and to truly examine relationships at the within-person level (Bolger & Laurenceau, 2013). We included time with respect to the repeated assessments per person as a fixed and random effect to control for potential reactivity effects.

Fourth, to investigate within-person associations between social partners, places, and activities with state expression, we ran variable-centered analyses and examined both bivariate and multivariate associations. To examine *bivariate within-person* associations, we conducted within-person correlations using the *rmcorr* package in R (Bakdash & Marusich, 2020). This allows detecting associations between each category of social partners, places, and activities (i.e., a total of 21 categories) with state expression that might otherwise be obscured or spurious due to aggregation. To examine *multivariate within-person* associations, we performed five random-intercept-random-slope models with state expression as the respective outcome. We included all 21 categories of variety in daily life simultaneously as predictor variables (see Tables S1 for bivariate correlations). The categories of social partners, places, and activities were dummy-coded (Nezlek, 2011). The *reference category* was the combination of the most frequently occurring categories of each situational feature: (a) being alone (social partners), (b) being indoor at home (places),^5^ and (c) working, studying, and volunteering^6^ (activities). The final model at Level 1 was structured as

$${\text{state}\;\text{expressiont}}_{\mathrm{ti}}=\beta_{0}\left(\text{reference}\;\text{category}\right)+u_{0i}+\beta_{1}\left({\text{social}\;\text{partner}}_{\mathrm{ti}}\right)+\ldots +\beta_{18}\left({\text{activity}}_{\mathrm{ti}}\right)+u_{19i}\left({\text{time}}_{\mathrm{ti}}\right)$$

where a person*’*s specific momentary state expression at measurement point *t* is modeled as a function of an intercept *β*_0_, representing the expected level of state expression when being alone, at home, and working, studying, and volunteering (reference category), which was allowed to vary between individuals (*u*_0*i*_), coefficients *β*_1_ to *β*_18_, reflecting the expected differences in momentary state expression for each social partner, place, and activity from the intercept, time (*β*_19_), which was allowed to vary between persons (*u*_19i_), and residual error *e*_*ti*_.

All multilevel analyses were performed using the *lme4* package in R (Bates & Fox, 2019). For all analyses, given the number of comparisons and large sample size, we employed a more stringent alpha level of .01 for discussion of the findings. Data, analysis code, and materials are available at the OSF repository and can be accessed at https://osf.io/bsv98.

## Results

### How Diverse is Daily Life in Terms of Social Partners, Places, and Activities?

Table 1 shows the descriptive statistics for all categories of social partners, places, and activities as well as variety scores. Note that the frequency with which social partners are met, places visited, and activities undertaken was constrained by the categories used. For example, some activities (e.g., working, studying) are undertaken daily by most individuals (except during vacations), while other activities (e.g., doing chores, errands) leave more room for variation within and across individuals. Participants were most likely to report being alone (38.0%), being at home (39.7%), and working, studying, or volunteering (44.8%). They showed between-person differences in average variety, as indicated by the broad range in the variety scores, from relatively low (0, average variety in daily social partners, places, and activities), to relatively high variety scores (2.03, average variety in daily places). The results also indicate between-person differences in daily variety. Generally, the between-person variance for daily variety was relatively low, with .16 for variety in social partners, .08 for variety in places, and .13 for variety in activities. That is, 84%–92% of the total variance related to differences within individuals. Descriptive statistics of and correlations between daily variety scores are shown in Tables S2–S4 and individual descriptive statistics are available at https://osf.io/bsv98.

**Table 1.** Descriptive Statistics of the Variety in Social Partners, Places, and Activities.

	Frequency or *M* (*SD*)^a^	% or Min, Max^a^
Social partners		
Alone	5392	38.0%
Partner	1246	8.8%
Family/children	1845	13.0%
Friends	1756	12.4%
Colleagues	2492	17.6%
Acquaintances	305	2.1%
Strangers	1154	8.1%
Daily variety^a^	.58 (.26)	0, 1.21
Average variety^a^	1.07 (.40)	0, 1.81
Places		
Indoor at home	5524	39.7%
Indoor at workplace	3382	24.3%
Indoor at a private place	630	4.5%
Indoor at a public place	991	7.1%
Outdoor at a private place	652	4.7%
Outdoor at a public place	1219	8.8%
In transit	1529	11.0%
Daily variety^a^	.61 (.25)	0, 1.21
Average variety^a^	1.09 (.50)	0, 2.03
Activities		
Working/studying/volunteering	5750	44.8%
Chores/errands	509	4.0%
Sport/movement	604	4.7%
Media	1344	10.5%
Doing nothing/relaxing/sleeping	2080	16.2%
Conversation/visit	1045	8.1%
Leisure	1513	11.8%
Daily variety^a^	.59 (.29)	0, 1.14
Average variety^a^	1.13 (.42)	0, 1.84

*Note. N* = 957–962, observations = 12,845–14,190.

### How Variable is the Expression of Personality States in Daily Life?

Table 2 depicts the descriptive statistics and variance decomposition for the state expression for each Big Five domain. Participants showed between-person differences in their within-person mean levels as well as in their amount of within-person variability as suggested by the respective ranges. Across the six days, participants showed the highest mean levels in openness (i*M*_*M*_ = 4.94), and the lowest in neuroticism (i*M*_*M*_ = 3.24). They varied most in their expression of extraversion (i*SD*_*M*_ = 1.35), and showed the smallest amount of variability in agreeableness (i*SD*_*M*_ = 1.01). This difference in variation may be related to the fact that the extraversion items (e.g., “quiet – talkative”) seem more sensitive to within-person variation than the agreeableness items (e.g., “distrustful – trusting”) and thus reflects a measurement issue. The between-person variance ranged from .16 (extraversion) to .26 (neuroticism and agreeableness), indicating that individuals varied more within themselves from assessment to assessment than from other people in each Big Five domain.

**Table 2.** Descriptive Statistics of the State Expression for the Big Five Domains.

State Expression	ICC	Within-Person Mean (i*M*)				Within-Person Variability (i*SD*)			
		*M*	*SD*	Min	Max	*M*	*SD*	Min	Max
Neuroticism	.26	3.24	0.83	1.00	6.14	1.16	0.40	0	2.83
Extraversion	.16	4.33	0.77	1.92	7.00	1.35	0.43	0	2.89
Openness	.23	4.94	0.73	1.50	7.00	1.02	0.39	0	2.77
Agreeableness	.26	4.84	0.75	2.00	7.00	1.01	0.40	0	2.32
Conscientiousness	.23	4.76	0.77	1.50	7.00	1.11	0.42	0	2.83

*Note. N* = 927–962. ICC: intraclass correlation; i*SD*: intraindividual standard deviation.

### How is Variety in Daily Life Related to the Expression of Personality States?

Table 3 shows the results of multilevel analyses linking daily variety scores with state expressions. At the between-person level, there were no significant links between variety in daily social partners and state expression. This indicates that the average variety in social partners across the study period was unrelated to average state expression. Greater variety in daily places was associated with more extraversion, indicating that participants who were in diverse places were also more extraverted. Finally, variety in activities was negatively associated with extraversion. That is, those individuals who were generally engaged in many different types of activities across the six days reported less extraversion than those people with less varied activities.

**Table 3.** Fixed Effects of Multilevel Modeling of Variety in Daily Life on State Expression.

Variable	State Expression									
	Neuroticism		Extraversion		Openness		Agreeableness		Conscientiousness	
	*b (SE)*	95% CI	*b (SE)*	95% CI	*b (SE)*	95% CI	*b (SE)*	95% CI	*b (SE)*	95% CI
Intercept	3.41** (.09)	[3.23, 3.59]	4.01** (.09)	[3.83, 4.17]	4.78** (.08)	[4.62, 4.93]	4.58** (.08)	[4.42, 4.74]	4.81** (.09)	[4.65, 4.98]
Daily variety in social partners (*between*)	.22 (.13)	[−.03, .48]	.03 (.12)	[−.12, .26]	−.04 (.11)	[.25, .17]	.00 (.11)	[−.22, .23]	.01 (.12)	[−.23, .24]
Daily variety in social partners (*within*)	−.01 (.04)	[−.08, .06]	.12* (.04)	[.03, .19]	.04 (.03)	[−.02, .10]	.01 (.03)	[−.06, .07]	.05 (.04)	[−.02, .12]
Daily variety in places (*between*)	−.03 (.13)	[.29, .23]	.41** (.12)	[.18, .64]	.20 (.11)	[−.02, .41]	.15 (.12)	[−.07, .37]	.16 (.12)	[−.08, .39]
Daily variety in places (*within*)	−.03 (.04)	[.11, .05]	.14* (.04)	[.05, .22]	.08 (.03)	[.01, .15]	.05 (.04)	[−.02, .12]	.15** (.04)	[.07, .22]
Daily variety in activities (*between*)	−.24 (.12)	[.48, −.00]	−.28* (.11)	[−.49, −.06]	−.02 (.10)	[−.22, .18]	.14 (.11)	[−.07, .35]	−.14 (.11)	[−.35 .07]
Daily variety in activities (*within*)	−.16** (.04)	[.23, −.08]	−.01 (.04)	[−.09, .06]	.00 (.03)	[−.06, .06]	.09* (.03)	[.02,.15]	−.13** (.03)	[−.19,−.05]
Time	−.01** (.00)	[−.02, −.01]	.02** (.00)	[.02, .03]	.01** (.00)	[.00, .01]	.01** (.00)	[.00, .01]	−.01** (.00)	[−.01, .00]

*Note. N* = 951, observations = 14,255. Estimates are unstandardized multilevel regression coefficients with standard errors in parentheses.

*p < .01. **p < .001.

A more complex picture emerged at the within-person level (Table 3). In contrast to the between-person findings, variety in social partners was significantly linked with the expression of extraversion. That is, on days when participants were with more different social partners than they normally were, they indicated more extraverted states. Furthermore, days with greater variety in places were related to higher levels in extraversion and conscientiousness within individuals. Finally, days with greater variety in activities than on average were associated with less neuroticism and conscientiousness, but with more agreeableness within individuals. In summary, we observed more significant associations between variety in daily life and state expression at the within-person level than at the between-person level.

### How Are Social Partners, Places, and Activities Related to the Expression of Personality States?

**Figure 1.**
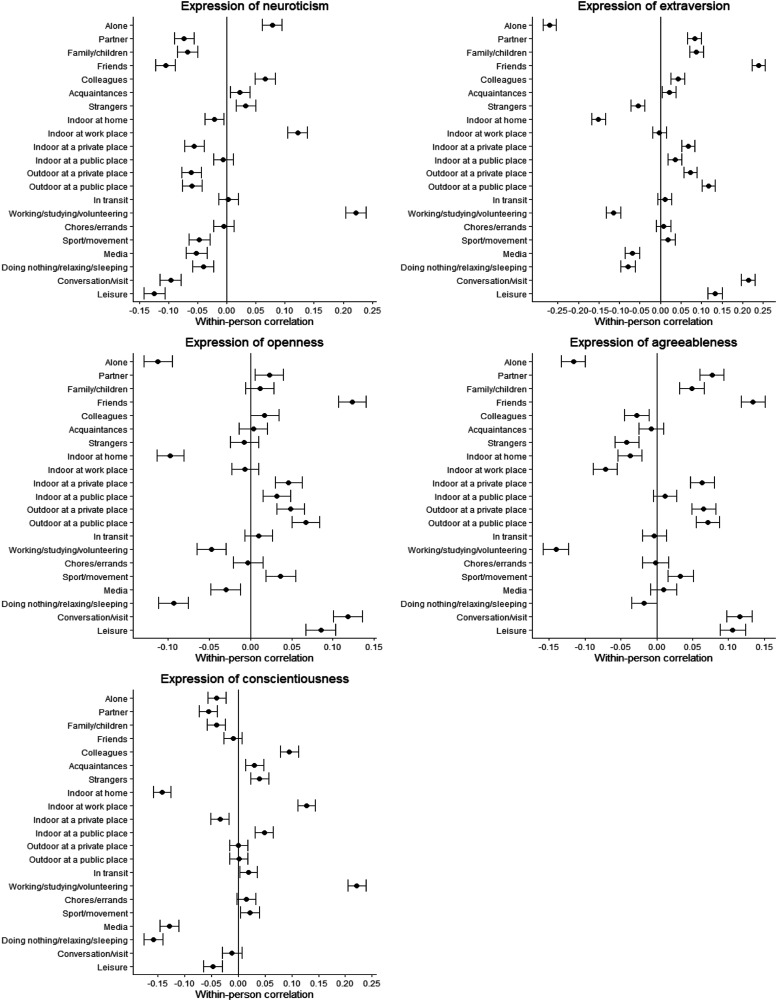
Bivariate within-person correlation between social partners, places, and activities with state expression including 95% confidence intervals for the coefficients.

To facilitate comparison with other studies, Figure 1 includes the bivariate within-person correlation coefficients between individual categories of the three situation cues and state expression, including 95% confidence intervals (CIs). Most situation-state associations were significantly associated, which indicates that participants varied in their state expressions depending on situational cues. For instance, when participants were alone they reported higher neuroticism, but lower extraversion, openness, agreeableness, and conscientiousness. A different pattern of state expressions emerged when participants were together with friends such that they reported lower levels in neuroticism and higher levels in extraversion, openness, and agreeableness expressions. Conscientiousness was not significantly related to situations in which individuals were with their friends. We also observed location-specific patterns. For example, when participants were at home they reported lower levels of extraversion, agreeableness, openness, and conscientiousness but higher levels of neuroticism. This may suggest that this specific place does not provide many opportunities for state expressions. In contrast, when participants were at the workplace they reported higher levels of neuroticism and conscientiousness, and lower levels of agreeableness and extraversion. Regarding associations with activities, we found that being engaged in work-related activities was associated with higher levels of neuroticism and conscientiousness, and at the same time lower levels of extraversion, openness, and agreeableness. Based on recent suggestions (Funder & Ozer, 2019), the significant within-person correlation coefficients ranged between negligible small (*r* = < .05) to medium-sized effects (*r* = .26; friends and extraversion).

Table 4 depicts the results of multivariate within-person associations using multilevel modeling. The intercept indicates the respective state expression when a person was alone, being indoor at home, and working, studying, or volunteering (combination of the three reference categories). Participants differed significantly from each other in their respective intercept for each of the state expression. The effects of different social partners, places, and activities are interpreted in relation to the intercept by either adding or subtracting the corresponding estimate. For instance, when an individual was with the partner instead of being alone, they were significantly less neurotic, but higher in extraversion, openness, and agreeableness. Compared to being alone, extraversion was significantly higher when being with strangers. Unlike the other states, conscientiousness was not significantly related to any social partners.

**Table 4.** Multilevel Modeling Fixed Effects of Social Partners, Places, and Activities on State Expression.

Variable	State Expression									
	Neuroticism		Extraversion		Openness		Agreeableness		Conscientiousness	
	*b (SE)*	*95% CI*	*b (SE)*	*95% CI*	*b (SE)*	*95% CI*	*b (SE)*	*95% CI*	*b (SE)*	*95% CI*
Intercept^a^	3.70** (.04)	[3.61, 3.78]	3.48** (.04)	[3.40, 3.56]	4.67** (.04)	[4 .60, 4.74]	4.46** (.04)	[4.39, 4.53]	4.90** (.04)	[4.83, 4.98]
Social partners										
Partner	−.23** (.05)	[−.32,−.14]	.71** (.05)	[.61, .81]	.19** (.04)	[.11, .27]	.35** (.04)	[.27, .43]	.00 (.04)	[−.09, .09]
Family/children	−.23** (.04)	[−.30, −.15]	.68* (.04)	[.60, .77]	.13** (.04)	[.06, .20]	.24** (.04)	[.17, .31]	.05 (.04)	[−.03, .13]
Friends	−.38** (.04)	[−.46, −.29]	1.10** (.05)	[1.01, 1.19]	.35** (.04)	[.28, .42]	.44** (.04)	[.37, .51]	−.07 (.04)	[−.15, .01]
Colleagues	−.19** (.04)	[−.26, −.11]	.58** (.04)	[.50, .67]	.16** (.04)	[.09, .22]	.17** (.04)	[.10, .23]	.01 (.04)	[−.07, .08]
Acquaintances	−.01 (.08)	[−.16, .15]	−.06 (.05)	[.28, .62]	.05 (.07)	[−.09, .19]	.08 (.07)	[−.06, .22]	.03 (.08)	[−.12, .18]
Strangers	.02 (.05)	[−.08, .12]	.19* (.07)	[−.17, .04]	−.02 (.04)	[−.11, .07]	−.05 (.04)	[−.14, .04]	.07 (.05)	[−.02, .16]
Places										
Indoor at workplace	.06 (.04)	[−.02, .14]	.27** (.05)	[.18, .36]	.10* (.04)	[.03, .17]	.06 (.04)	[−.01, .14]	.23** (.04)	[.15, .31]
Indoor at a private place	−.23** (.06)	[−.35, −.11]	.24** (.07)	[.11, .37]	.20** (.05)	[.10, .31]	.21** (.05)	[.11, .32]	.01 (.06)	[−.11, .13]
Indoor at a public place	−.07 (.05)	[−.18, .03]	.24** (.06)	[.13, .35]	.20** (.05)	[.11, .29]	.13* (.05)	[.04, .22]	.29** (.05)	[.19, .39]
Outdoor at a private place	−.08 (.06)	[−.19, .03]	.33** (.06)	[.21, .46]	.21** (.05)	[.11, .31]	.20** (.05)	[.10, .30]	.23** (.06)	[.12, .34]
Outdoor at a public place	−.12 (.05)	[−.22, −.03]	.53** (.05)	[.43, .63]	.26** (.04)	[.17, .34]	.22** (.04)	[.13, .30]	.13* (.05)	[.04, .22]
In transit	.00 (.04)	[−.09, .09]	.41** (.05)	[.31, .50]	.21** (.04)	[.14, .29]	.14** (.04)	[.06, .22]	.28** (.04)	[.19, .36]
Activities										
Chores/errands	−.35** (.06)	[−.47, −.22]	.45** (.09)	[.06, .32]	−.01 (.06)	[−.12, .10]	.13 (.06)	[.02, .24]	−.24** (.06)	[−.36, −.12]
Sport/movement	−.51** (.06)	[−.62, −.39]	.16 (.07)	[.04, .29]	.16* (.05)	[.05, .26]	.26** (.05)	[.16, .37]	−.16* (.06)	[−.27, −.05]
Media	−.53** (.04)	[−.61, −.44]	.10 (.05)	[.01, .20]	.03 (.04)	[−.04, .11]	.26** (.04)	[.19, .34]	−.68** (.04)	[−.76, −.59]
Doing nothing/relaxing/sleeping	−.42** (.04)	[−.49, −.34]	.04 (.04)	[−.04, .13]	−.14** (.03)	[−.21, −.07]	.15** (.03)	[.08, .21]	−.65** (.04)	[−.72, −.58]
Conversation/visit	−.55** (.05)	[−.65, −.45]	.81** (.05)	[.71, .92]	.34** (.04)	[.26, .43]	.42** (.04)	[.33, .50]	−.32** (.05)	[−.41, −.22]
Leisure	−.62** (.04)	[.71, −.54]	.52** (.05)	[.42, .61]	.23** (.04)	[.15, .31]	.38** (.04)	[.30, .45]	−.40** (.04)	[−.48, −.32]
Time	.00 (.00)	[−.01, .00]	.01** (.00)	[.01, .02]	.00 (.00)	[.00, .00]	.00 (.00)	[.00, .01]	−.00 (.00)	[.00, .00]

*Note. N* = 950, observations = 12,346. Estimates are unstandardized multilevel regression coefficients with standard errors in parentheses.

^a^The intercept is the reference category and includes the simultaneous occurrence of the categories of “being alone,” “indoor at home,” and “working, studying, or volunteering.”

*p < .01. **p < .001.

Various places were associated with state expression. For instance, compared to being at home, being either inside or outside at private or public places, or in transit was related to higher extraversion, openness, agreeableness, and conscientiousness (except for a nonsignificant link between being indoor at a private place and conscientiousness). Spending time at the workplace was not associated with neuroticism, whereas being indoor at a private place was negatively related to neuroticism.

Finally, activities showed multiple significant associations with state expression. Compared to the work and study reference group, participants exhibited lower neuroticism and conscientiousness in all other activities. Specifically, during sports activities, conversation, or leisure activities, individuals showed lower neuroticism and conscientiousness, but higher extraversion (except for sports activities), openness, and agreeableness than when they were working. Doing nothing was associated with lower neuroticism, openness, and conscientiousness, but higher agreeableness compared to work-related activities. Taken all together, for some, but not all state expressions, being with different social partners, spending time at diverse places, and engaging in different activities contributed to the variability of state expressions from moment to moment.^7^

## Discussion

The present study examined the role of variety in social partners, places, and activities concerning the expression of personality states in daily life. Overall, the results highlight that variety in daily life can be considered an important individual differences variable that can vary within and between persons. The findings show that greater variety in the way people live their daily lives is associated with the way people express their state-related behaviors, thoughts, and feelings, and thus contribute to a better understanding how personality states manifest dynamically across diverse situations in daily life.

### Variety in Daily Life and Expression of Personality States

The first key finding is that daily life in terms of social partners, places, and activities is characterized by a substantial amount of variety. The descriptive results indicate that the three investigated situation cues showed a similar amount of variety within and across days. In terms of frequency with respect to the social partner categories, being alone and being with colleagues were mentioned most often. Consistent with Matz and Harari (2021), we found that home and the workplace (campus) are the most frequently visited places. The category of “working, studying, or volunteering” was the most frequently indicated category of activities, followed by “doing nothing, relaxing, or sleeping”. Furthermore, participants differed in the ways their lives are shaped by variety. Some individuals mostly spent time with the same social partner, were at the same place, and showed the same activity, whereas others were with various social partners, spent time at diverse places, and were involved in a wide array of activities across the six consecutive days. These results are consistent with previous research (e.g., Fingerman et al., 2020; Weber et al., 2020). In extending previous work, this study modeled daily variety longitudinally across six consecutive days. We found evidence that participants’ daily lives vary in variety from day to day. In fact, we evidenced a substantial amount of within-person variation in variety across social partners, places, and activities over time, indicating that variety in situation cues can change from day to day. Some days are homogeneous and less diverse, whereas other days are diverse in terms of social partners, places, and activities. Possible reasons for the variability in variety could be daily stressors (e.g., arguments, work deadlines; Almeida, 2005) and positive experiences, fluctuations in one’s daily well-being (Lee et al., 2018), or daily physical health constraints (Weber et al., 2020), which may limit how varied a day is arranged. As noted earlier, the description of frequency and variation within and between individuals in exposure to different situation cues is limited by the categories used. For instance, some places (e.g., indoor at work) are typically visited on a regular basis, while other places (e.g., outdoor at a private place) leave more room for variation within and across individuals.

The second key finding is that the expression of personality states is characterized by a substantial amount of variability. Indeed, we found sizable variation both within and between individuals in expressing personality states. This replicates previous work (e.g., Fleeson, 2001, 2007; Fleeson & Gallagher, 2009; Sherman et al., 2015). The ICCs in this study ranged from .16 to .26 (mean ICC of .23), suggesting that, on average, 23% of the variation in the state expression was due to between-person factors. Other studies reported slightly higher between-person variability (e.g., 35%; Fleeson & Gallagher, 2009; 36%; Sherman et al., 2015), which may be partly related to differences in samples, measurements, and study designs. Overall, substantial variation in state expression and in variety in daily life is an important prerequisite for linking state expression with diversity scores.

### How Variety in Daily Life Is Related to the Expression of Personality States

The main focus of this study was to examine how variety in social partners, places, and activities is related to state expression within and between individuals. Using a bivariate approach, we found that greater daily variety was associated with more state expression in some states. In the following, we discuss the results along the three cues of situations.

Findings with respect to variety in social partners suggest that on days with more than average variety, participants showed more extraversion within individuals. This finding complements previous research showing that individuals who were in a situation with higher sociality, a psychological situational characteristic (measured with the item “Social interaction is possible or required”), behaved more extravertedly than they normally would (Sherman et al., 2015), and that higher sociality predicts later expression of extraversion (Rauthmann et al., 2016). Variety in social partners provides opportunities and constraints to express extraversion differently depending on how many different interaction partners (e.g., friends, colleagues, strangers) are present (e.g., friends, colleagues, strangers). To express extraversion, social opportunities and triggering situations, respectively, are needed (Wrzus & Roberts, 2017). For example, meeting colleagues at work, spending time with several friends during leisure time, or meeting strangers when being in transit are social situations that may trigger extraverted behavior. Spending time alone or only with a partner does not require more effort to exhibit extraverted behavior.

Agreeableness, which like extraversion contains important social aspects, was not significantly related to variety in daily social partners. It is possible that extraversion, which has a positive affective signature in addition to a social one, is more contextually malleable and more responsive to situational aspects than agreeableness (Robinson, 2009). On the other hand, the results suggest that people express agreeableness regardless of a broad or limited range of daily social interaction partners. But as noted earlier, the extraversion and agreeableness items do not appear to be similarly sensitive to variability, so this difference may be due to that as well.

Variety in places was positively associated with the expression of extraversion and conscientiousness within individuals. That is, on days with more diverse places visited than average, participants showed more expressions in extraversion and conscientiousness. Similar to social contexts, different places (e.g., indoor at work or in transit) and frequent changes of locations (e.g., from home to office, from office to library) present a variety in opportunities and constraints for expressing extraversion and conscientiousness in a given situation, and thus promote or inhibit the expression of the states. Previous research found that people behaved more conscientiously than they otherwise would in situations in which the psychological situation characteristic of duty (measured with the item “The job must get done”) was higher (Sherman et al., 2015). Moreover, situations in which duty was higher was found to have a small spillover effect on later conscientiousness expression (Rauthmann et al., 2016). The association between variety in places and the expression of extraversion was also evidenced at the between-person level, suggesting that those participants who reported more variety in places visited across the entire study period, were those who also reported higher average levels of extraversion states.

Variety in activities was related to lower neuroticism and conscientiousness and higher agreeableness at the within-person level. For instance, days with higher than average variety in activity were associated with less expression of neuroticism and less expression of conscientiousness. It is possible that a greater repertoire of activities includes more activities eliciting positive reactions (e.g., relaxing effects, increased well-being), which, in turn, might lower neuroticism. A similar idea underlies behavioral activation as a therapeutic intervention to treat depression (Dimidjian et al., 2011). Behavioral activation is designed to help clients resume various activities that are important to them or that they have enjoyed in the past. Furthermore, a greater variety in daily activities seems to undermine conscientious behaviors and at the same time promote a stronger expression of agreeableness. Some activities (e.g., doing nothing, relaxing, or sleeping) do not require conscientiousness, whereas other activities (e.g., working, learning, or volunteering) appear to require conscientiousness. Finally, while extraversion was unrelated to variety in activities within-person, greater variety was associated with lower averaged extraversion at the between-person level.

### How Social Partners, Places, and Activities Are Related to the Expression of Personality States

Using a multivariate approach, we zoomed into daily life and examined within-person associations between all social partners, places, and activities with state expression. A more fine-grained, multivariate analysis is necessary to better understand the complex relationships between situation cues and state expression (“if-then” associations; Mischel & Shoda, 1995; Noftle & Gust, 2019; Rauthmann et al., 2016). In the following, we organize the discussion along the three situation cues.

The results related to social partners suggest that participants showed different patterns of state expression as a function of proximity to interaction partners. When participants were with close partners (i.e., partner, family/children, friends, colleagues) rather than being alone, they reported lower levels of neuroticism and higher levels of extraversion, openness, and agreeableness. This pattern of state expression did not hold true when participants were with less close partners (i.e., acquaintances or strangers). A similar differential result was observed in relation to affective experiences (Chui et al., 2014). Moreover, Sherman et al. (2015) have shown that situations with higher sociality are associated with more extraverted behavior in individuals, while the current findings further qualify this relationship, suggesting for future research that proximity to social partners may be a potential moderator of this association. One possible explanation could be that such a pattern of state expression may prove functional for maintaining and strengthening important close relationships (Leary, 2007; Yao & Moskowitz, 2015). These associations can be reinforced either by oneself (e.g., reinforcement through positive emotions) and/or by social interaction partners (e.g., reinforcement through smiling) in response to these state expressions (Wrzus & Roberts, 2017).

We found a consistent pattern of associations across all places. That is, when participants were away from home, they showed significantly more extraversion, openness, and agreeableness (except for agreeableness, which was unrelated to work location). This finding was also observed in recent research (Matz & Harari, 2021). A plausible explanation is that an individual’s home represents a quiet refuge to regain energy, whereas other places involve stimulating opportunities for these state expressions. Of all places, home has a particularly strong symbolic and psychological significance, representing a unique place where a person’s past, present, and future self is reflected and comes to life (Graham et al., 2015). Relatedly, it might be that state expressions are driven by social norms inherent to places. While one’s home as a private place offers the greatest freedom of choice, in other places, social norms may play a stronger role for the state expressions (Church et al., 2010; Krämer, 1996), which may result in different state expressions outside the home. For example, the motivation to adhere to the norms of sociability in a place may lead to a temporary increase in extraversion.

The results regarding daily activities showed a clear picture in terms of the expression of neuroticism, agreeableness, and conscientiousness, which could be interpreted as adaptation processes to different demands of diverse activities (Paulus & Martin, 1988). For instance, participants showed higher scores on neuroticism and conscientiousness expression, but more agreeableness (except for a nonsignificant association with doing chores, errands) in work-related activities, similar to previous findings (Minbashian et al., 2010; Wilson et al., 2017). It may be that during work-related activities, individuals tend to process information in a way that promotes negative states (i.e., stress, anxiety) (Rouse et al., 2020), leading to increased neuroticism. As mentioned earlier, engaging in various activities might be a promising way to reduce the experience of neuroticism in everyday life. Further research is needed to replicate the links between activity variety and neuroticism. High levels of conscientiousness and low levels of agreeableness at work could be due to motivational processes (e.g., get tasks done; McCabe & Fleeson, 2016) and play a role in the accomplishment of difficult, important, and urgent tasks (Wood et al., 2019). This finding complements the findings of Sherman et al. (2015), who demonstrated a positive within-person association between the situation perception that a task must be completed and the expression of conscientious behavior.

Concerning extraversion and openness, a mixed picture emerged. For example, while conversation and leisure activities were positively associated with higher levels of openness compared to the reference category of working or studying, doing nothing or relaxing was the only category of activities associated with lower levels of openness. It could be that these latter activities facilitated unwinding from daily stress, for which openness (i.e., being curious, open-minded) might not be beneficial. Future research is needed to look at possible mechanisms underlying these situation-personality expression associations.

### Implications, Limitations, and Future Directions

The findings reported here have implications for theoretical accounts that focus on the role of situational factors and individual processes in the expression of personality states. Specifically, they highlight the role that variability in daily life can play in personality state expression. First, although the current results do not allow causal conclusions regarding the effects of situations on state expressions, the present findings contribute to the TESSERA model (Wrzus & Roberts, 2017) by suggesting that variety in daily life can be conceptualized as a trigger for state expressions, in addition to situational cues and their perceived psychological meaning. Second, our findings align with WTT (Fleeson & Jayawickreme, 2015) in that the situational factor of variety in daily life explains individual differences in the density distribution of repeatedly rated personality states across different situations. Third, the present results also contribute to situation research (Rauthmann & Sherman, 2020) by showing that the within-person associations between variety in situation cues and state expression may constitute an important person-situation phenomenon. Finally, the present findings may be informative for the Nonlinear Interaction of Person and Situation (NIPS; Blum et al., 2018) model, which distinguishes between “strong” and “weak” situations and persons. The NIPS model assumes that situations with a moderate level of affordance are the weakest situations that exhibit the greatest variability in state expression between different individuals. It also assumes that strong individuals exhibit less variability in their state expression between different situations, while weak individuals show greater variability. Future research is needed to examine different levels of affordances associated with variety in social partners, locations, and activities and their potential nonlinear associations with the expression of personality states.

The present research is limited in ways that should promote future research. Some limitations are related to assessment. For instance, the measures of situation cues partly included categories that naturally differed in breadth (e.g., “colleagues” may comprise several different people, whereas “partner” typically refers to one particular person) or categories with multiple meanings (e.g., “working, studying, or volunteering”). Although our analyses only captured variety between different categories within one situation cue (e.g., social partners) and not within a category (e.g., chores, errands), these limitations may have led to some over- or underestimation of variety in daily life. To gain more detailed insight into variety in daily life, it would be valuable in future research to use measures with categories that are comparable in breadth. It is also possible that variability in state expression was influenced by the fact that some items of the very short adjective-based measure of personality states captured variation better than others. Future work is needed to develop established measures with items that are similarly sensitive to variability. Another limitation refers to the fact that only self-report measures were used. One promising approach would be to combine self-reports with sensing assessment techniques that allow the study of objective technology-based behavioral indicators of everyday contexts (Beierle et al., in press; Rüegger et al., 2020). Likewise, a multi-method assessment approach will help to further expand our knowledge of the links between variety in daily life and state expression.

Other limitations are related to the study design. The present work is correlational and thus does not allow causal conclusions regarding the effects of situation cues on state expressions and vice versa. However, we believe that it is important to first systematically identify correlation patterns between a variety of situation cues and state expression in a large sample between and within individuals. Future research should investigate causal relationships using experimental designs. Moreover, as the present study period was restricted to one week, intensive-longitudinal studies with longer time intervals and measurement burst designs are needed (e.g., Pfund, Hofer, Allemand, & Hill, 2022). In the present study, we focused on how variety in daily life was associated with the expression of states. However, as discussed earlier, in addition to situational factors, various individual processes may also play an important role as mechanisms in state expression (e.g., cognitive and motivational processes; Fleeson & Jayawickreme, 2015). For example, recent research demonstrated that state expressions help to satisfy psychological needs (Lindner et al., 2021), which in turn may promote state expression. Future studies of variety in daily life must consider situational factors and individual processes. Finally, the present sample was restricted to a single country, consisted mostly of students, and was largely homogenous in terms of cultural identity and socioeconomic status. As such, future research needs to examine whether findings generalize to other contexts, particularly ones with lower socioeconomic status.

## Conclusion

The current findings advance our understanding of how variety in daily life is associated with the expression of personality states by identifying how different situation cues of social partners, places, and activities contribute to individuals’ state expressions. The study thus highlights the need for conceptualizing and measuring state expressions in the context of situational variety to contribute to a better understanding of how people behave, think, and feel in their daily lives. Thus, the results enrich the growing field of personality dynamics and processes by further demonstrating that variation in state expression within individuals in relation to situation cues is a meaningful phenomenon of personality. This study may inspire future research to investigate why people differ in the way they live their daily lives.

## Supplemental Material

Supplemental Material - How Is Variety in Daily Life Related to the Expression of Personality States? An Ambulatory Assessment Study Supplemental Material for How is Variety in Daily Life Related to the Expression of Personality States? An Ambulatory Assessment Study by Stefanie Lindner, Mirjam Stieger, Dominik Ruegger, Tobias Kowatsch, Christoph Flückiger, Matthias Mehl, and Mathias Allemand in European Journal of Personality.

## Notes

1 Other researchers have used the term diversity. For example, Weber et al. (2020, p. 435) defined diversity in daily life as “the experience of varying situations during or across days (e.g., different social contacts or activities). The term diversity is used synonymously with variety in different categories of a phenomenon (...).” In this study, we use the term variety because diversity is often used in the context of inclusion of people regardless of race, ethnicity, age, gender, religion, sexual orientation, etc.

2 A detailed report of the study design, sample size calculation, recruitment process, and measures can be found in the Stieger et al. (2018).

3 One previous paper used a subsample of this dataset with the daily assessments (*N* = 316 Android user) to examine associations between self-reported personality states and smartphone data (Rüegger et al., 2020).

4 To test for possible differences between the 1–7 response scale used and the original 1-100 scale, we examined the correlations between repeated personality expressions and average variety of social partners, places, and activities. Differences in correlations (Δ*r*s) between the two response scales were smaller than .008, suggesting that the results were virtually the same.

5 In their analyses of the associations between places and personality expression, Matz and Harari (2021) also used “home” as reference category.

6 Note that working, studying, volunteering represents one activity category (see section daily life measures).

7 We also tested whether age (Noftle & Gust, 2019) and weekdays versus weekend affected state expressions. Specifically, we included a dichotomous variable in the multilevel models that split weekdays (0 = Monday to Friday) from weekend (1 = Saturday) to control for potential temporal effects. Tables S5–S6 show that the inclusion of age (grand-mean centered) and weekend as covariates in the multilevel models produced similar findings for both sets of multilevel models (MLM) (for the five MLM testing the associations between daily variety and state expression, as well as for the five MLM testing associations between the context and activity categories and state expression). In the five MLM testing the associations between daily variety and state expression, weekend did not have an effect on the variety-state expression associations, but had a significant effect on state expressions. More specifically, individuals showed less neuroticism on the weekend (i.e., Saturday).
