# Supplementary material for: How Is Variety in Daily Life Related to the Expression of Personality States? An Ambulatory Assessment Study
Source: Eur J Pers. 2023 Jan 9;38(2):172–88. doi: 10.1177/08902070221149593 (PMC13029511; doi:10.1177/08902070221149593)
Supplement: Supplemental Material - How Is Variety in Daily Life Related to the Expression of Personality States? An Ambulatory Assessment Study [file sj-pdf-1-erp-10.1177_08902070221149593.pdf]

Table S1

Bivariate Correlations among Features of Situations

	1	2	3	4	5	6	7	8	9	10	11	12	13	14	15	16	17	18	19	20
2 Partner	-.24**																			
3 Family	-.30**	-.12**																		
4 Friends	-.29**	-.12**	-.15**																	
5 Colleagues	-.36**	-.14**	-.18**	-.17**																
6 Acquaintances	-.12**	-.05**	-.06**	-.05**	-.07**															
7 Strangers	-.23**	-.09**	-.12**	-.11**	-.14**	-.04**														
8 Work	-.04**	-.17**	-.20**	-.08**	.37**	.06**	.02													
9 Chores	.02	.02	.05**	-.03**	-.08**	-.02**	.07**	-.18**												
10 Sport	.01	-.01	-.03*	.05**	-.06**	.00	.04**	-.20**	-.05**											
11 Media	.14**	.04**	.05**	-.09**	-.14**	-.04**	-.02	-.31**	-.07**	-.08***										
12 Doing nothing	.12**	.10**	.07**	-.09**	-.16**	-.04**	-.03**	-.40**	-.09**	-.10***	-.15**									
13 Conversation	-.19**	.05**	.11**	.20**	-.05**	.03**	-.05**	-.27**	-.06**	-.07***	-.10**	-.13**								
14 Leisure	-.06**	.07**	.08**	.12**	-.12**	-.02	-.01	-.33**	-.07**	-.08***	-.13**	-.16**	-.11**							
15 Indoor at home	.32**	.08**	.21**	-.17**	-.33**	-.08**	-.22**	-.26**	-.06**	-.11***	.29**	.28**	-.08**	-.02						
16 Indoor at work place	-.18**	-.14**	-.21**	-.03**	.54**	.06**	-.03**	.59**	-.12**	-.12***	-.19**	-.22**	-.13**	-.21**	-.46**					
17 Indoor at a private place	-.10**	.15**	.04**	.09**	-.06**	.00	-.07**	-.12**	-.03**	-.04***	.00	.02	.20**	.03**	-.18**	-.12**				
18 Indoor at a public place	-.13**	-.02**	-.06**	.13**	.01	.07**	.14**	.05**	.07**	.10***	-.08**	-.10**	.02	.00	-.22**	-.16**	-.06**			
19 Outdoor at a private place	-.04**	.04**	.10**	.04**	-.07**	-.01	-.06**	-.12**	-.01	.00	-.03**	.04**	.13**	.08**	-.18**	-.13**	-.05**	-.06**		
20 Outdoor at a public place	-.05**	.00	-.03**	.13**	-.06**	.01	.05**	-.18**	.10**	.25***	-.10**	-.06**	.07**	.16**	-.25**	-.18**	-.07**	-.09**	-.07**	
21 In transit	-.01	-.04**	-.06**	-.02	-.10**	-.02	.32**	-.15**	.15**	.06***	-.01	-.01	.02	.10**	-.29**	-.20**	-.08**	-.10**	-.08**	-.11**

Note. *$p < .01$. **$p < .001$.

Table S2

Pearson Correlation and Descriptive Statistics of Daily Variety in Social Partners

	Day 1	Day 2	Day 3	Day 4	Day 5	Day 6
Day 2	.11*					
Day 3	.09*	.21**				
Day 4	.13**	.23**	.29**			
Day 5	.15**	.07	.20**	.15**		
Day 6	.01	.04	.13	.05	.07	
i*M*	.65	.68	.69	.55	.62	.45
i*SD*	.38	.38	.38	.44	.40	.40
Min	.00	.00	.00	.00	.00	.00
Max	1.39	1.39	1.39	1.46	1.39	1.39

Note. N = 346-954. *SD*: standard deviation.
*$p < .01$. **$p < .001$.

Table S3

Pearson Correlation and Descriptive Statistics of Daily Variety in Activities

	Day 1	Day 2	Day 3	Day 4	Day 5	Day 6
Day 2	.14**					
Day 3	.12*	.14**				
Day 4	.10*	.29**	.21**			
Day 5	.07	.05	.00	.03		
Day 6	.02	.02	.06	.08	.11	
M	.64	.67	.69	.52	.67	.55
SD	.39	.40	.39	.48	.41	.43
Min	.00	.00	.00	.00	.00	.00
Max	1.39	1.39	1.39	1.46	1.39	1.39

Note. N = 338-954. *SD*: standard deviation.
*$p < .01$. **$p < .001$.

Table S4

Pearson Correlation and Descriptive Statistics of Daily Variety in Places

	Day 1	Day 2	Day 3	Day 4	Day 5	Day 6
Day 2	.12**					
Day 3	.06	.13**				
Day 4	.13*	.07	.18**			
Day 5	.04	.01	.13*	.12*		
Day 6	.03	.03	.03	.03	.06	
M	.64	.71	.68	.65	.63	.33
SD	.39	.40	.38	.40	.42	.39
Min	.00	.00	.00	.00	.00	.00
Max	1.39	1.39	1.39	1.39	1.39	1.10

Note. *N* = 338-954. *SD*: standard deviation.
**p* < .01. ***p* < .001.

Table S5

Multilevel Modeling Fixed Effects of Daily Variety on State Expressions with Covariates

Variable	State Expressions									
	Neuroticism		Extraversion		Openness		Agreeableness		Conscientiousness	
	b (SE)	95% CI	*b (SE)*	95% CI	*b (SE)*	95% CI	*b (SE)*	95% CI	*b (SE)*	95% CI
Intercept	3.42 ** (.09)	[3.23, 3.60]	4.00** (.09)	[3.82, 4.17]	4.77** (.08)	[4.61, 4.93]	4.57** (.08)	[4.41, 4.74]	4.79** (.09)	[4.63, 4.96]
Daily variety in social partners (*between*)	.16 (.13)	[-.09, .42]	.09 (.12)	[-.14, .32]	.02 (.11)	[-.19, .23]	.05 (.11)	[-.17, .27]	.07 (.12)	[-.15, .30]
Daily variety in social partners (*within*)	-.02 (.04)	[-.09, .07]	.12* (.04)	[.03, .19]	.04 (.03)	[-.02, .10]	.02 (.03)	[-.05, .07]	.04 (.04)	[-.02, .11]
Daily variety in activities (*between*)	-.25 (.12)	[-.49, -.02]	-.27 (.11)	[-.47, -.07]	-.01 (.10)	[-.21, .18]	.15 (.10)	[-.06, .34]	-.14 (.11)	[-.35, .07]
Daily variety in activities (*within*)	-.16** (.04)	[-.23, -.08]	-.01 (.04)	[-.08, .06]	.00 (.03)	[-.05, .06]	.08* (.03)	[.02, .15]	-.12** (.03)	[-.18, -.05]
Daily variety in places (*between*)	.00 (.13)	[-.26, .26]	.38* (.12)	[.14, .60]	.16 (.11)	[-.05, .37]	.12 (.11)	[-.10, .34]	.12 (.12)	[-.11, .35]
Daily variety in places (*within*)	.04 (.04)	[-.12, .04]	.15* (.04)	[.04, .23]	.09* (.03)	[.01, .15]	.06 (.04)	[-.02, .11]	.13** (.04)	[.07, .22]
Age	.01** (.00)	[-.01, -.00]	.01** (.00)	[.00, .02]	.02** (.00)	[.00, .02]	.01** (.00)	[.01, .01]	.02** (.00)	[.01, .02]
Day[a]	-.16* (.06)	[-.27, -.04]	.14 (.07)	[.01, .27]	.11 (.05)	[.01, .21]	.12 (.05)	[.01, .22]	-.14 (.06)	[-.25, -.03]
Time	-.01** (.00)	[-.02, -.01]	.02** (.00)	[.02, .03]	.01** (.00)	[.00, .01]	.00 (.00)	[.00, .01]	-.01** (.00)	[-.01, .00]

Note. *N* = 951, observations = 14,242. Estimates are unstandardized multilevel regression coefficients with standard errors in parentheses. [a] 0: weekday (Monday to Friday), 1: weekend (Saturday).

**p* < .01. ***p* < .001.

Table S6

Multilevel Modeling Fixed Effects of Situational Aspects on State Expressions Including Covariates

Variable	State Expressions									
	Neuroticism		Extraversion		Openness		Agreeableness		Conscientiousness	
	b (SE)	95% CI	*b (SE)*	95% CI	*b (SE)*	95% CI	*b (SE)*	95% CI	*b (SE)*	95% CI
Intercept [a]	3.69** (.04)	[3.61, 3.77]	3.48** (.04)	[3.40, 3.56]	4.67** (.04)	[4.60, 4.74]	4.47** (.04)	[4.39, 4.54]	4.91** (.04)	[4.83, 4.99]
Social partners										
Partner	-.23** (.05)	[-.32, -.14]	.71** (.05)	[.61, .80]	.18** (.04)	[.10, .26]	.34** (.04)	[.26, .42]	-.00 (.04)	[-.09, .08]
Family/children	-.22** (.04)	[-.30, -.14]	.68** (.04)	[.59, .76]	.12** (.04)	[.05, .19]	.24** (.04)	[.17, .31]	.05 (.04)	[-.03, .12]
Friends	-.38** (.04)	[-.46, -.30]	1.11** (.05)	[1.02, 1.20]	.36** (.04)	[.29, .43]	.44** (.04)	[.37, .52]	-.06 (.04)	[-.14, .01]
Colleagues	-.19** (.04)	[-.26, -.11]	.58** (.04)	[.50, .67]	.16** (.04)	[.09, .23]	.17** (.04)	[.10, .24]	.01 (.04)	[.07, .08]
Acquaintances	-.01 (.08)	[-.16, .15]	.45** (.09)	[.28, .62]	.05 (.07)	[-.09, .19]	.08 (.07)	[-.05, .22]	.03 (.08)	[-.12, .18]
Strangers	.02 (.05)	[-.08, .11]	-.06 (.05)	[-.16, .05]	-.02 (.04)	[-.10, .07]	-.05 (.04)	[-.13, .04]	.07 (.05)	[-.02, .17]
Activities										
Chores/errands	-.34** (.06)	[-.46, -.22]	.18* (.07)	[.05, .32]	-.02 (.06)	[-.13, .09]	.12 (.06)	[.01, -.23]	-.25** (.06)	[-.37, -.13]
Sport/movement	-.50** (.06)	[-.62, -.39]	.16 (.07)	[.03, .29]	.15* (.05)	[.05, .26]	.26** (.05)	[.15, .36]	-.16* (.06)	[-.28, -.05]
Media	-.53** (.04)	[-.61, -.44]	.10 (.05)	[.01, .20]	.03 (.04)	[-.04, .11]	.26** (.04)	[.18, .34]	-.68** (.04)	[-.76, -.59]
Doing nothing/ relaxing/sleeping	-.42** (.04)	[-.49, -.34]	.04 (.04)	[-.04, .12]	-.14** (.03)	[-.21, -.08]	.15** (.03)	[.08, .21]	-.65** (.04)	[-.72, -.58]
Conversation/visit	-.55** (.05)	[-.64, -.45]	.81** (.05)	[.70, .91]	.34** (.04)	[.26, .43]	.42** (.04)	[.33, .50]	-.33** (.05)	[-.42, -.23]
Leisure	-.62** (.04)	[-.71, -.53]	.51** (.05)	[.42, .60]	.23** (.04)	[.15, .30]	.37** (.04)	[.30, .45]	-.40** (.04)	[-.49, -.32]
Places										

Indoor at work place	.07 (.04)	[-.01, .15]	.26** (.05)	[.17, .35]	.09 (.04)	[.01, .16]	.06 (.04)	[-.02, .13]	.22** (.04)	[.14, .30]
Indoor at a private place	-.23** (.06)	[-.35, -.11]	.24** (.07)	[.11, .37]	.20** (.05)	[.10, .31]	.21** (.05)	[.10, .32]	.01 (.06)	[-.10, .13]
Indoor at a public place	-.07 (.05)	[-.17, .03]	.24** (.06)	[.12, .35]	.19** (.05)	[.10, .29]	.13* (.05)	[.04, .22]	.29** (.05)	[.19, .39]
Outdoor at a private place	-.07 (.06)	[-.01, .04]	.32** (.06)	[.20, .45]	.20** (.05)	[.10, .31]	.20** (.05)	[.10, .30]	.23** (.06)	[.11, .34]
Outdoor at a public place	-.12 (.05)	[-.21, -.02]	.53** (.05)	[.42, .63]	.25** (.04)	[.17, .34]	.21** (.04)	[.13, .30]	.12* (.05)	[.03, .21]
In transit	.00 (.04)	[-.08, .09]	.41** (.05)	[.31, .50]	.21** (.04)	[.13, .29]	.14** (.04)	[.06, .22]	.27** (.04)	[.19, .36]
Age	-.01** (.00)	[-.02, -.01]	.01** (.00)	[.01, .02]	.02** (.00)	[.01, .02]	.01** (.00)	[.01, .02]	.02** (.00)	[.01, .02]
Day[b]	-.02 (.06)	[-.14, .09]	-.00 (.06)	[-.13, .12]	.07 (.05)	[-.03, .17]	.02 (.05)	[-.08, .12]	-.09 (.06)	[-.20, .02]
Time	-.00 (.00)	[-.01, .00]	.01** (.00)	[.01, .02]	.00 (.00)	[.00, .01]	.00 (.00)	[-.00, 01]	-.00 (.00)	[.01, .00]

Note. *N* = 950, observations = 12,346. Estimates are unstandardized multilevel regression coefficients with standard errors in parentheses.

[a] The intercept is the reference category and includes the simultaneous occurrence of the situational factors alone, working/studying/volunteering, and indoor at home; [b] 0: weekday (Monday to Friday), 1: weekend (Saturday).

**p* < .01. ***p* < .001.
